# Study protocol of a 4- parallel arm, superiority, community based cluster randomized controlled trial comparing paper and e-platform based interventions to improve accuracy of recall of last menstrual period (LMP) dates in rural Bangladesh

**DOI:** 10.1186/s12889-018-6258-z

**Published:** 2018-12-10

**Authors:** Shumona Sharmin Salam, Nazia Binte Ali, Ahmed Ehsanur Rahman, Tazeen Tahsina, Md. Irteja Islam, Afrin Iqbal, Dewan Md. Emdadul Hoque, Samir Kumar Saha, Shams El Arifeen

**Affiliations:** 1 Maternal and Child Health Division, icddr,b, Dhaka, 1212 Bangladesh; 2 Department of Microbiology, Dhaka Shishu (Children’s) Hospital, Dhaka, 1207 Bangladesh

**Keywords:** Gestational age, LMP, Recall, Preterm birth, Bangladesh, Mobile phone, M-health

## Abstract

**Background:**

Gestational age (GA) is a key determinant of newborn survival and long-term impairment. Accurate estimation of GA facilitates timely provision of essential interventions to improve maternal and newborn outcomes. Menstrual based dating, ultrasound based dating, and neonatal estimates are the primarily used methods for assessing GA; all of which have some strength and weaknesses that require critical consideration. Last menstrual period (LMP) is simple, low-cost self-reported information, recommended by the World Health Organization for estimating GA but has issues of recall mainly among poorer, less educated women and women with irregular menstruation, undiagnosed abortion, and spotting during early pregnancy. Several studies have noted that about 20–50% of women cannot accurately recall the date of LMP. The goal of this study is therefore to improve recall and reporting of LMP and by doing so increase the accuracy of LMP based GA assessment in a rural population of Bangladesh where antenatal care-seeking, availability and utilization of USG is low.

**Method:**

We propose to conduct a 4- parallel arm, superiority, community based cluster randomized controlled trial comparing three interventions to improve recall of GA with a no intervention arm. The interventions include (i) counselling and a paper based calendar (ii) counselling and a cell phone based SMS alert system (iii) counselling and smart-phone application. The trial is being conducted among 3360 adolescent girls and recently married women in Mirzapur sub-district of Bangladesh.

**Discussion:**

Enrolment of study participants continued from January 24, 2017 to March 29, 2017. Data collection and intervention implementation is ongoing and will end by February, 2019. Data analysis will measure efficacy of interventions in improving the recall of LMP date among enrolled participants. Results will be reported following CONSORT guideline.

The innovative conventional & e-platform based interventions, if successful, can provide substantial evidence to scale-up in a low resource setting where m-Health initiatives are proliferating with active support from all sectors in policy and implementation.

**Trial registration:**

ClinicalTrials.gov NCT02944747. The trial has been registered before starting enrolment on 24 October 2016.

**Electronic supplementary material:**

The online version of this article (10.1186/s12889-018-6258-z) contains supplementary material, which is available to authorized users.

## Background

Accurate estimation of gestational age (GA) is critical for providing optimal care during pregnancy, childbirth and postnatal period [1, 2]. It is essential for tracking fetal growth and appropriate administration of live saving interventions related to pre-term births such as antenatal corticosteroids therapy (ACS), Kangaroo Mother Care (KMC), tocolytics, and continuous positive airway pressure [1, 2]. The importance of accurate estimation of GA is paramount in Bangladesh as Prematurity/Low birth weight (LBW) accounts for 13% of all newborn deaths [3–5]. The national guideline of Bangladesh also recommends using ACS for women with threatened preterm deliveries between 24 and 34 weeks of GA and KMC for preterm/LBW babies [6–9].

Three methods of GA estimation are primarily used - menstrual based dating (LMP), ultrasonography (USG) and neonatal assessment, all of which have some strengths and weaknesses [10]. USG, based on biometric measurements of the fetus, is considered the gold standard when it is done before 20 weeks of gestation [11]. However, in low and middle income countries (LMIC) like Bangladesh, majority of the first antenatal care (ANC) contacts happen after 20 weeks of Gestation [3, 12]. Out of those who have an USG performed within 20 weeks, there are issues with validity and reliability of measurements due to the poor quality of equipment and inadequate skill of the health care providers [12, 13]. Moreover, USG is expensive, and the availability is limited in resource poor settings [12]. Neonatal assessments are based on standardized scoring systems of physical and neuromuscular maturity of the newborns. These are, however, less precise then USG, and also require skilled personnel [14]. LMP is a simple, low-cost method recommended by the World Health Organization [15]. However, several studies have shown that approximately 15–45% of pregnant women are unable to recall their LMP accurately [16–18]. The accuracy of LMP-recall is negatively influenced by digit preference, duration of the recall period, irregularity or individual variations in the length of the menstrual cycle, conception during the period of lactational amenorrhea, preconception amenorrhea and implantation bleeding [16–20]. The accuracy is further compromised by late presentation for the first antenatal care contact and minimal skills of the ANC providers in probing for an accurate LMP date. Reliance on LMP-recall as the only method for estimating GA may therefore result in overestimation of GA at the extremes of gestation, which is more common in women with no or low education and those living in poverty [16, 21]. Despite the limitations, studies conducted in Bangladesh and Guatemala has suggested LMP to be the preferred method for determining GA in low-resource settings [12, 22].

We therefore aim to determine the efficacy of three interventions targeted to improve LMP-recall among women in rural Bangladesh.

### Goals and objectives

The goal of this study is to improve the accuracy of LMP-recall based estimation of (GA). Through this trial we aim to determine the efficacy of paper based LMP dating calendar and e-platform based interventions (SMS based system and smart phone applications) in improving accuracy of LMP-recall among adolescent girls and recently married women in rural Bangladesh.

### Trial design

We propose to undertake a 4 - parallel arm, superiority, community based cluster randomized controlled trial comparing a no intervention arm with three intervention arms. The four arms are as follows:

- Arm 1: No intervention arm
- Arm 2: Counselling and a paper based calendar
- Arm 3: Counselling and a mobile-phone based SMS alert system
- Arm 4: Counselling and smart-phone application

**Fig. 1:**
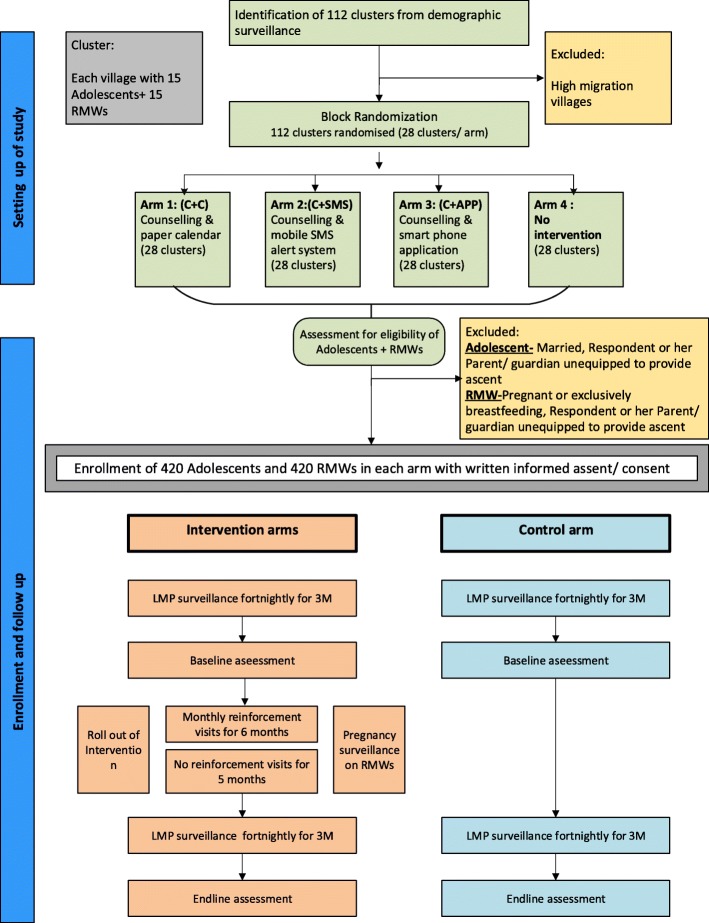
Study design (created by authors)

The trial was designed following SPIRIT guideline (Additional file 1). The design and randomization process has been illustrated in Fig. 1.

## Methods

### Study setting

**Fig. 2:**
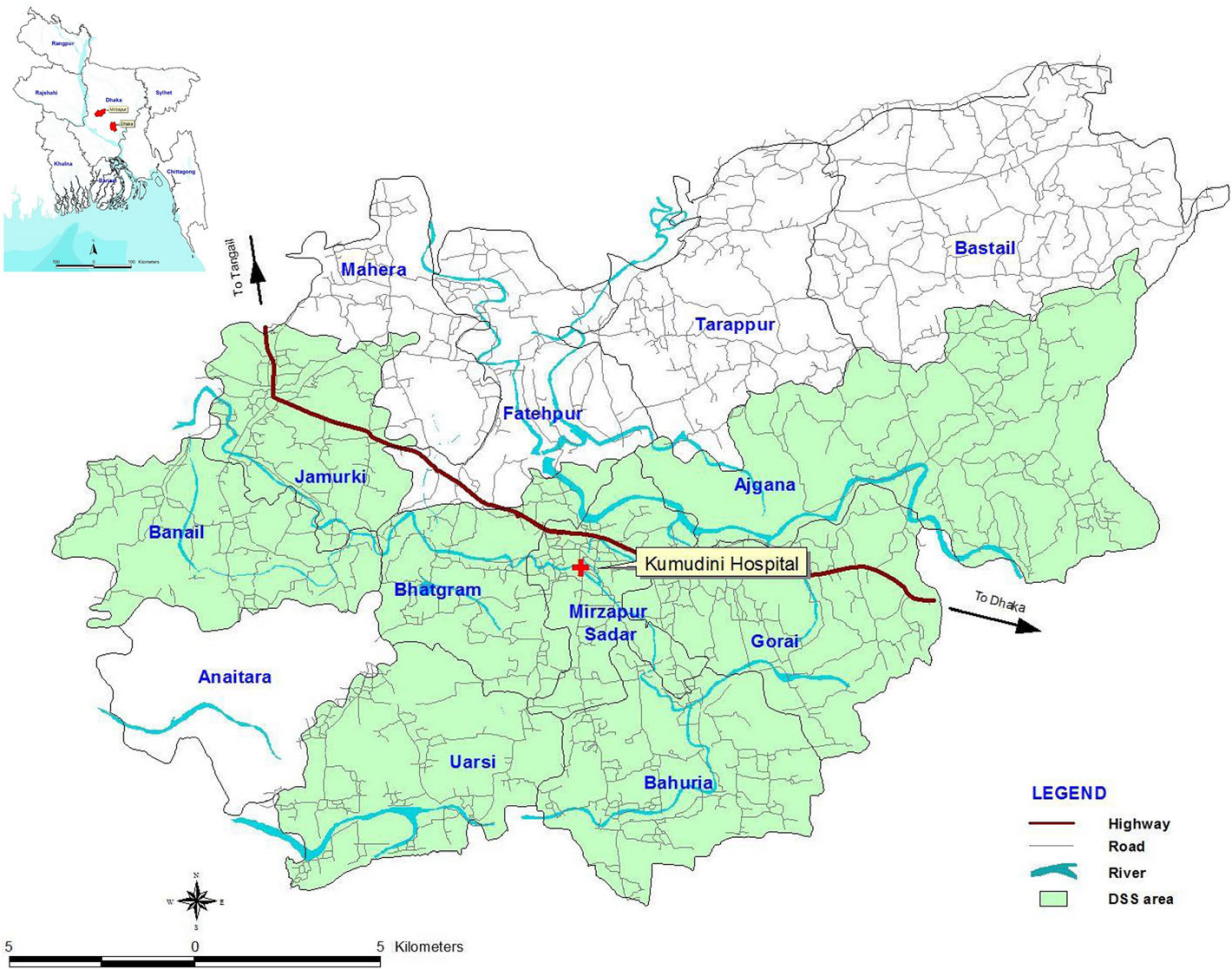
Study area map (created by authors)

The study is being carried out in Mirzapur sub-district of Tangail district located at the northwest of Dhaka city, the capital of Bangladesh. Administratively, Mirzapur has 13 unions (the lowest administrative body with an average population of around 30,000), with 219 villages covering an area of 375 sq. km and a population of 3, 37, 496 (Fig. 2).

Since 2007, icddr,b has been running a demographic surveillance system (DSS) in 10 unions of Mirzapur covering 293,691 with a four monthly round. The trial will be implemented in the DSS area for ease of identification and enrolment of study participants.

### Participants

Adolescent girls (15–17 years of age) and recently married women (within past two and half years) with no or a single child residing in the selected villages has been enrolled to participate in the study. Table 1 shows the inclusion and exclusion criteria for enrolment.

**Table 1:** Eligibility criteria

	Adolescents	Recently married women
Inclusion criteria	• Permanent resident of the study area (Adolescents who are usual residents of the households and living or intend to live in the area for 6 or more months)	• Permanent resident of the study area(recently married women who are usual residents of the households living or intend to live in the area for 6 or more months)
	• Aged between 15 and 17 years at the time of enrolment	• Aged < 45 years at the time of enrolment
	• Unmarried at the time of enrolment	• Married within the past two and half years with no or a single child
	• Parent or legal guardian is present at the time of recruitment and is willing to give assent for the adolescent’s participation	• Legal guardian is present at the time of recruitment and is willing to give assent for the participation of the recently married women aged < 18 years
Exclusion criteria	• Not applicable	• Pregnant or exclusively breastfeeding at the time of recruitment
	• Participant is mentally/physically challenged to provide consent	• Participant is mentally/physically challenged to provide consent

### Study arms

The study arms and interventions being tested in this study are described below:

- *Arm 1-no intervention arm*: In the no intervention arm, study participants follow their usual practices to recall LMP start and end dates; if any.
- *Arm 2- counselling and a paper based calendar*: This intervention entails counselling and documentation of menstrual bleeding dates on a paper based calendar. The topics of counselling include normal menstrual cycle length, abnormal bleeding, menstrual hygiene and importance of remembering and recording LMP dates. In addition, a free especially designed paper-based calendar is provided to the participant who prospectively records menstrual bleeding and spotting dates in each month. Likewise, if anybody forgets to record, she keeps a retrospective record of it in the subsequent month. Female counsellors from the study team conducted the individual education sessions.
- *Arm 3- counselling and a cell-phone based SMS alert system*: This intervention entails counselling and a cell-phone based SMS alert system. Counselling is similar to the calendar arm. Cell-phones are provided free of cost to the study participants who shares their first date of menstrual period every month to a designated number through an SMS. A automated-system is designed to record the dates from the SMSs. An SMS reminder is sent 3 days after the tentative start date of the cycle (as will be obtained from a LMP surveillance). If the user still does not input the start date, 2 other reminders are sent at an interval of 2 days. There is provision for sending unlimited LMP start dates in 1 month with restrictions to enter future dates. All the instructions are provided in Bangla. The charge of SMS for reporting LMP dates is free for the user.
- *Arm 4- counselling and smart-phone application*: Participants of this intervention arm received counselling and a free smart-phone with an application for recording menstruation dates and an inbuilt reminder system. Counselling is done on similar topics as the calendar arm. The smart phone based application is a password protected, colour-coded system for recording start date and end date of each menstrual cycle. The app is in Bangla (local language). Participants record start date and end date of their LMP each month in the application. There are provisions for entering dates for multiple cycles in a month and retrospective recording of dates (in case the user forgot to enter their previous cycles).

### Screening, consent & enrolment of study participants

The study participants (adolescent girls and recently married women) has been selected using DSS database. Participants selected from the DSS data base were approached by the field research staffs (surveillance workers) in a chronological way through household visits and enrolled in the study after getting written informed consent/ assent. Enrolment continued till the required sample size was achieved.

Two sets of consents/assents were taken from the study participants in the three intervention arms; one for participation in the intervention and the other for data collection. In the no intervention arm, consent/assent was obtained only for participation in data collection. Assent was taken from the legal guardians of all participants below 18 years of age. All the consent/assent forms are in Bangla for better understanding in local context. Details of the consent forms(purpose of the study, activities, risk, benefit, confidentiality, anonymity, future use of information, right not participate etc.) were read out and explained to study participants by the field research staffs before taking consent. Those who agreed to participate in the study signed two copies of consent/assent forms (thumb print were taken in case of illiterate participants); a copy of consent/assent was given to the study participant and the other one kept at icddr,b central office for record keeping.

### Delivering & monitoring the interventions

The roll out of interventions were arm specific. Female counsellors provided individual counselling to the enrolled study participants and handed over the specific interventions (paper-based calendars, mobile phones and smart phones) with adequate demonstration on how to record LMP-dates using the tools.

Adoption of interventions were reinforced by follow-up visits. For the first 2 months, each study participant received weekly household visit followed by biweekly visits in the subsequent 2 months. During the follow up visits, each participant were requested to record their LMP dates in the given tool and troubleshooting (device issues, balance & internet connectivity problems, forgot how to use the tools etc.) were done if needed. Participants are discontinued from the study in case of pregnancy (recently married women), marriage (adolescent girls), and permanent migration out of the study area, withdrawal of consent to participate etc.

Quality of intervention implementation are monitored by independent field research supervisors and investigators.

### Outcome measures

The primary outcome of this trial is the accuracy of LMP-recall dates. Accuracy of LMP-recall is defined as participants who will able to recall their start date of LMP within ±1 day of actual start date for their last menstrual cycle.

The secondary outcome of the trial is the certainty of LMP-recall dates. Participants will be asked to mention their level of certainty regarding their LMP-recall dates using the Likert scale ranging from 1 to 4 (highly certain, almost certain, somewhat certain, uncertain). Certainty will be defined if the participant are ‘highly certain’ regarding their recall for the last menstrual cycle.

Additionally we will also validate the LMP-dates recorded through the intervention tools with dates obtained from surveillance.

### Sample size

Sample size has been calculated to observe a minimum of 30% relative change in the primary outcome measure. We used STATA (comparison of two proportions in the presence of clustering effect) for sample size calculation. Assuming that in the rural context of Bangladesh approximately 55% of women can accurately recall LMP (at baseline without any intervention) [13], to measure a 30% relative change in recall as an effect of the intervention, we will require 252 participants from 28 clusters per arm (5% level of significance and 90% power). Accounting for 15% refusal and 20% loss to follow-up, the adjusted sample size was 420 adolescents and 420 recently married women per arm (Table 2).

**Table 2:** Sample distribution

Number of samples					
	Arm1: Control arm	Arm2: Counselling & calendar (C + C)	Arm3: Counselling & SMS (C + S)	Arm4: Counselling & smart phone application	Total
Recently married women	420	420	420	420	1680
Adolescent girls	420	420	420	420	1680

### Participant timeline

The study will be conducted for 34 months. Enrolments continued till achievement of sample size. Interventions will be continued for 12 months. Detailed of the study timeline has been illustrated in Additional file 2.

### Study cluster formation, randomization & assignment of intervention

The trial administrator for this study was responsible for cluster formation and randomization of study clusters in the study arms using block randomization in stata.

At first, DSS data has been used to identify the required number of adolescent girls recently married women from the 10 unions after exclusion of high migration villages. One hundred twelve individual clusters were formed using GIS (geographic information system) technique with at least 15 adolescent girls and 15 recently married women per cluster. Buffer spaces between clusters (geographic spaces like river, roads, wet land, village boundary etc) were ensured during this exercise to reduce spill over effect.

All the field staffs (including managers and workers) & study participants were unaware of the intervention allocation during enrolment & baseline data collection. Recruitment of field research staffs were arm specific. Separate teams are responsible for data collection and intervention implementation for ensuring allocation concealment.

### Methods of data collection, management and analysis

Data for this study is being collected in the following ways:

(i) *LMP surveillance*

Actual LMP-dates of the enrolled study participants are being collected at two time points through 3-month long surveillances.. The baseline LMP surveillance was done before the start of implementation of interventions whilst the endline will be done before the end of implementation. A tab-based LMP surveillance tool (specially designed for this study), is used by field surveillance workers to collect this information through fortnightly rounds. No other information will be elicited from the participants during LMP-surveillance.

(ii) *LMP surveys*

A baseline survey was conducted among enrolled adolescents and recently married women in all arms before the start of the implementation of interventions. Face-to face interviews were done by trained data collectors using a structured questionnaire to elicit the required information. Participants were asked to recall their past 3 months LMP dates and their level of certainty regarding the LMP-recall dates. Information regarding demographic characteristics (age, sex, education, occupation), household’s socioeconomic profiles (housing status, assets, monthly income, ownership of mobile phone & accessibility to mobile phone), menstrual history were also collected. Recently married women were additionally asked regarding the use of contraceptive, pregnancy and birth outcomes. A similar survey will be conducted at endline.

(iii) *Data* ***e*** *xtraction from the intervention tools*

Data is extracted regularly from calendar records, SMS and smart-phones. The users of SMS arm sends their LMP dates via SMS whereas users of the smart-phone upload their data via the internet connected to the mobile-phone. For participants using the calendar, data collectors collect the information from the enrolled household every 2 months.

### Data management and analysis

#### Data management

Data quality will be ensured through rigorous training of the data collectors, refresher trainings and frequent spot checks by field supervisors. All the paper based data will be checked for completeness and will be entered into Microsoft access data base. An experienced data manager will be in charge of monitoring data quality. Any inconsistency will be resolved through rechecking the forms or by revisiting the households if possible. A real time online monitoring system (dash board) has been designed for quick and timely availability of information on intervention adherence, and data collection summary in intervention arms.

#### Data analysis

A participant flow diagram will be prepared following CONSORT 2010 guideline. Primary data analysis will be done on intention to treat basis. Analyses will be conducted at the individual level, and will be adjusted for cluster randomization.

For the primary outcome analysis, we will first examine the proportion of women accurately reporting LMP-dates at baseline and at endline. Differences in differences method will be used to ascertain the change. The analysis will be adjusted for confounders such as education, socio-economic status, and length of recall by bivariate analysis. Additionally, we will explore the change in mean and standard deviation of the difference between LMP-recall dates collected through LMP surveys and LMP-dates collected through LMP surveillance.

We will also validate the tools by comparing the distribution of LMP-dates recorded through the intervention tools with actual LMP-dates collected through LMP surveillance. Skewness and kurtosis tests of distribution of two LMP dates (recorded LMP and actual LMP) will be compared.

Stata® software version 10.1 (2008; Stata Corporation, College Station, TX, USA) will be used for all analyses.

#### Data safety monitoring plan (DSMP)

We anticipate no risk of interventions. Validity and integrity of the data is being ensured by appropriate research design, use of pretested and validated tools for data collection and by quality assurance.

#### Data confidentiality and archiving

Privacy, anonymity and confidentiality of data is strictly being maintained. In order to protect the safety of participants, all trial related information is being kept confidential and stored securely at the central office in icddr,b. Coded identification is used to anonymise and depersonalise the data. The linking code, electronic data files and paper forms are stored in a separate location under password protections or lock and key. Access to the data will be limited to the small number of individuals including the investigators, statisticians, quality control team and audit.

#### Dissemination policy

The trial results will be communicated and published irrespective of the outcome of the trial upon approval from all the investigators, implementing organizations and funding bodies.

### Project update

Enrolment of study participants continued from January 24, 2017 till March 29, 2017. Baseline data collection commenced from April 2017 and was completed in October, 2017. Implementation of interventions started from October 2017 and are currently ongoing. Endline data collection will start from mid-September, 2018.

## Discussion

This trial is expected to generate evidence on efficacy of calendar, SMS and smart phone application system for improving accuracy of LMP-recall in rural settings. Although calendar, or menstrual dairies has already been tried in clinical settings for recording menstrual dates, public health implication of using these tools for recording LMP is yet to be explored [16, 23]. The nationwide coverage of mobile phone networks, relatively cheaper tariff and increasing use of smart phone among general population including the poor, presets m-health as a potential solution to address this issue. [3, 23–25].

## Additional files

Additional file 1: Spirit checklist. (DOC 112 kb)

Additional file 2: Study timeline. (PDF 182 kb)

## Abbreviations

ANC: Antenatal care
BMGF: Bill Melinda Gates foundation
DSS: Demographic surveillance system
GA: Gestational age
icddr,b: International centre for diarrhoeal disease research, Bangladesh
LMIC: Low and middle income countries
LMP: Last menstrual period
USG: Ultrasonography

## References

[1] Wilcox AJ, Weinberg CR, Basso O (2011). On the pitfalls of adjusting for gestational age at birth. Am J Epidemiol.

[2] Ananth CV (2007). Menstrual versus clinical estimate of gestational age dating in the United States: temporal trends and variability in indices of perinatal outcomes. Paediatr Perinat Epidemiol.

[3] National Institute of Population Research and Training (NIPORT), Mitra and Associates, and ICF International (2015). Bangladesh Demographic and Health Survey 2014: Key Indicators.

[4] Blencowe H, Cousens S, Oestergaard MZ, Chou D, Moller AB, Narwal R (2012). National, regional, and worldwide estimates of preterm birth rates in the year 2010 with time trends since 1990 for selected countries: a systematic analysis and implications. Lancet.

[5] Liu L, Johnson HL, Cousens S, Perin J, Scott S, Lawn JE (2012). Global, regional, and national causes of child mortality: an updated systematic analysis for 2010 with time trends since 2000. Lancet.

[6] UNICEF (2013). Committing to child survival: a promise renewed. Progress report 2013.

[7] Government of Bangladesh (GoB). Bangladesh every newborn action plan. Dhaka: Ministry of Health and Family Welfare, Government of Bangladesh; 2016.

[8] World Health Organization. Kangaroo mother care: a practical guide. Geneva: World Health Organization; 2003.

[9] Lawn JE, Mwansa-Kambafwile J, Horta BL, Barros FC, Cousens S (2010). Kangaroo mother care’ to prevent neonatal deaths due to preterm birth complications. Int J Epidemiol.

[10] Lynch CD, Zhang J (2007). The research implications of the selection of a gestational age estimation method. Paediatr Perinat Epidemiol.

[11] Hutchon DJ (1999). Routine ultrasound is the method of choice for dating pregnancy. Br J Obstet Gynaecol.

[12] Persson LÅ, Arifeen S, Ekström E-C, Rasmussen KM, Frongillo EA, Team MS (2012). Effects of prenatal micronutrient and early food supplementation on maternal hemoglobin, birth weight, and infant mortality among children in Bangladesh: the MINIMat randomized trial. JAMA.

[13] Rosenberg RE, Ahmed AS, Ahmed S, Saha SK, Chowdhury MA, Black RE (2009). Determining gestational age in a low-resource setting: validity of last menstrual period. J Health Popul Nutr.

[14] Pereira AP, Dias MA, Bastos MH, da Gama SG, Leal Mdo C (2013). Determining gestational age for public health care users in Brazil: comparison of methods and algorithm creation. BMC Res Notes.

[15] WHO. Home-based maternal records. Guidelines for development, adaptation and evaluation. Geneva: World Health Organization; 1994.

[16] Wegienka G (2005). Baird DD. A comparison of recalled date of last menstrual period with prospectively recorded dates. J Womens Health (Larchmt).

[17] Waller DK, Spears WD, Gu Y, Cunningham GC (2000). Assessing number-specific error in the recall of onset of last menstrual period. Paediatr Perinat Epidemiol.

[18] Van Oppenraaij RH, Eilers PH, Willemsen SP, van Dunne FM, Exalto N, Steegers EA (2015). Determinants of number-specific recall error of last menstrual period: a retrospective cohort study. BJOG.

[19] Gjessing HK, Skjaerven R, Wilcox AJ (1999). Errors in gestational age: evidence of bleeding early in pregnancy. Am J Public Health.

[20] Jukic AM, Baird DD, Weinberg CR, McConnaughey DR, Wilcox AJ (2013). Length of human pregnancy and contributors to its natural variation. Hum Reprod.

[21] Hoffman CS, Messer LC, Mendola P, Savitz DA, Herring AH, Hartmann KE (2008). Comparison of gestational age at birth based on last menstrual period and ultrasound during the first trimester. Paediatr Perinat Epidemiol.

[22] Neufeld LM, Haas JD, Grajéda R, Martorell R (2006). Last menstrual period provides the best estimate of gestation length for women in rural Guatemala. Paediatr Perinat Epidemiol.

[23] Smith-DiJulio K, Mitchell ES, Woods NF (2005). Concordance of retrospective and prospective reporting of menstrual irregularity by women in the menopausal transition. Climacteric.

[24] Watterson JL, Walsh J, Madeka I. Using mHealth to improve usage of antenatal care, postnatal care, and immunization: a systematic review of the literature. Biomed Res Int. 2015;2015:1-9. PMID: 26380263. 10.1155/2015/153402 PMC4561933 26380263

[25] Ahmed T, Bloom G, Iqbal M, Lucas H, Rasheed S, Waldman L, Khan AS, Islam R, Bhuiya A. E-health and M-health in Bangladesh: opportunities and challenges. Brighton: IDS; 2014.

