# Supplementary material for: Study protocol of a 4- parallel arm, superiority, community based cluster randomized controlled trial comparing paper and e-platform based interventions to improve accuracy of recall of last menstrual period (LMP) dates in rural Bangladesh
Source: BMC Public Health. 2018 Dec 10;18:1359. doi: 10.1186/s12889-018-6258-z (PMC6288958; doi:10.1186/s12889-018-6258-z)
Supplement: Supplementary file 2 — Study timeline. (PDF 182 kb) [file 12889_2018_6258_MOESM2_ESM.pdf]

Additional file 1: Schedule of enrolment, interventions, and assessments of the study

	2016								2017												2018												2019	
**Activities**	**M**	**J**	**J**	**A**	**S**	**O**	**N**	**D**	**J**	**F**	**M**	**A**	**M**	**J**	**J**	**A**	**S**	**O**	**N**	**D**	**J**	**F**	**M**	**A**	**M**	**J**	**J**	**A**	**S**	**O**	**N**	**D**	**J**	**F**
	1	2	3	4	5	6	7	8	9	10	11	12	13	14	15	16	17	18	19	20	21	22	23	24	25	26	27	28	29	30	31	32	33	34
IRB Approval		↔	↔	↔	↔	↔																												
Development of survey and surveillance tools		↔	↔	↔	↔	↔	↔	↔	↔	↔	↔	↔	↔	↔																				
Development of applications (phone based)													↔	↔	↔	↔	↔																	
Development of phone based SMS system													↔	↔	↔	↔	↔																	
Development of Calendar and IEC material																↔	↔																	
Enrollment and consent of study participants									↔	↔	↔																							
LMP surveillance (Baseline)												↔	↔	↔	↔																			
Baseline survey																↔	↔	↔																
Intervention implementation																		↔	↔	↔	↔	↔	↔	↔	↔	↔	↔	↔	↔					
With follow up visits																		↔	↔	↔	↔	↔	↔											
Without follow up visits																								↔	↔	↔	↔	↔	↔					
Pregnancy Surveillance																							↔	↔	↔	↔	↔	↔	↔	↔	↔			
LMP surveillance (Endline)																													↔	↔	↔			
Endline survey																																↔	↔	
Final reporting																																	↔	↔
